# Morphological and Biochemical Effects on the Skeletal Muscle of Ovariectomized Old Female Rats Submitted to the Intake of Diets with Vegetable or Animal Protein and Resistance Training

**DOI:** 10.1155/2016/9251064

**Published:** 2016-01-14

**Authors:** Glaucia Figueiredo Braggion, Elisabete Ornelas, Jurema Carmona Sattin Cury, Natália Edviges Alves Lima, Rita C. Aquino, Fernando Luiz Affonso Fonseca, Laura Beatriz Mesiano Maifrino

**Affiliations:** ^1^Laboratory of Morphological and Immunohistochemical Studies, Universidade São Judas Tadeu, Brazil; ^2^Postgraduate Program (Stricto Sensu), Ph.D. Course in Physical Education, Universidade São Judas Tadeu, Brazil; ^3^Biological Sciences Department, Federal University of São Paulo, Diadema, SP, Brazil; ^4^Laboratory of Clinical Analysis, ABC Medical School, Santo André, SP, Brazil

## Abstract

*Introduction*. Sarcopenia is a process characterized by reduction in protein mass and muscle strength with increasing age, especially in the postmenopausal period, resulting in functional limitations and with great impact on the physical autonomy of the elderly.* Objective*. To evaluate the effects of diets with vegetable proteins (VP) or animal proteins (AP) associated with resistance training (RT) on the structural and biochemical parameters of the medial gastrocnemius muscle in Wistar rats with sarcopenia.* Methods*. An experimental model with ovariectomized rats was used to induce sarcopenia and resistance training. The histochemical technique was used for the typing of muscle fibers, the cross-sectional area of myocytes, and volume densities of myocytes and interstitium; the technique of Picrosirius stain was used to highlight the collagen fibers.* Results*. The VP diet was not able to minimize the effects of sarcopenia in the medial gastrocnemius of sedentary animals and when associated with RT, it promoted maintenance of the CSA, attenuating the atrophy of type IIB fibers in the medial gastrocnemius. The AP diet in sedentary animals protected the type I fibers. When combined with RT, the AP promoted muscle remodeling, with reduction in volume density of type I and IIA fibers, and increase of IIB fibers, together with an increase in collagen volume density.* Conclusion*. The data suggest a tendency to better results of hypertrophy in animal groups that consumed the AP diet, even the sedentary animals, although more evident in those trained.

## 1. Introduction

Due to the growth of life expectancy, according to the World Health Organization, the number of people classified as elderly has increased gradually [1]. Sarcopenia is a morphological change typical of aging [2] and characterized by a chronic and progressive process of 3–8% reduction of protein muscle mass per decade after the age of 30 years [3]. It affects 30% of individuals over 60 years of age and over 50% of those older than 80 years [4, 5].

Some mechanisms described in the literature as associated with the sarcopenia process are emphasized, such as hormonal factors, metabolism of proteins and their cellular signaling; voluntary or imposed reduction of the level of physical activity [6]; and protein malnutrition and reduced anabolic efficiency of protein intake [7–11], mitochondrial dysfunction [12], apoptosis of myocytes [13], and neurodegenerative diseases [14]. In women, menopause is a key factor in determining sarcopenia because of the hormone reduction involved in the muscle mass maintenance process that occurs in this phase of life [15].

Although the protein synthesis (the muscle synthesis in particular) is determined by a diverse range of factors, physical activity stimulation promotes significant responses of this synthesis if there is nutritional support for it. In this case, the dietary amino acid profile and the origin of protein (animal or vegetable) are a fundamental prerequisite [15–17].

The content of essential amino acids in food, the protein digestibility, and the distribution of these amino acids throughout the day are factors that also affect the result of protein synthesis stimulated by exercise [18]. The intake of carbohydrate with protein also appears to influence the protein synthesis capacity in the elderly, making the process more difficult due to inhibition of insulin-induced synthesis, unlike what happens with young adults [19–21].

Physical activity, especially resistance training, has been described in the literature as a determining factor for maintenance of muscle mass and reduction of intramuscular fat accumulation in the elderly [15, 22]. Thus, resistance exercise can be an important strategy to maintain muscle mass in postmenopausal women [23].

The objective of this study was to evaluate the effects of eating diets with vegetable or animal protein associated with resistance training on the structural and biochemical parameters of the medial gastrocnemius muscle in Wistar rats with sarcopenia.

## 2. Materials and Methods

### 2.1. Animals and Groups

This study was submitted to the Ethics Committee of Universidade São Judas Tadeu and approved under number A-00610/2010. A total of 30 females rats (*Rattus norvegicus* albinos - Rodentia Mammalia), Wistar, aged 21 days of life, were used. Animals were kept under controlled ambient conditions of temperature (23°C) and light (12-hour light/dark cycle) until they were aged 14 months, with free access to water supply, and the standard chow offered for laboratory animals (NUVILAB CR1, produced by NUVITAL Nutrientes LTDA, Curitiba, PR), composed of protein exclusively from vegetable sources.

From six months after ovariectomy, the animals were divided into six groups:

 CVS: sedentary, nonovariectomized, vegetable protein diet (*n* = 5).
 VOS: sedentary, ovariectomized, vegetable protein diet (*n* = 5).
 VOT: trained, ovariectomized, vegetable protein diet (*n* = 5).
 CAS: sedentary, nonovariectomized, animal protein diet (*n* = 5).
 AOS: sedentary, ovariectomized, animal protein diet (*n* = 5).
 AOT: trained, ovariectomized, animal protein diet (*n* = 5).

### 2.2. Resistance Training Protocol

The experimental protocol of physical training and diet started when the rats were 14 months old and eight months after ovariectomy. The equipment used was a vertical staircase made of wood with iron steps. The equipment is 110 cm high with 80° inclination and has a box on the top to accommodate the animals. The overload during resistance training was in the form of lead weights attached to the tail of the animal in the proximal portion.

All groups were submitted to a prior period of adaptation to the training protocol for a week, without any added load, in order to reach the rest area at the top of the stairs. The procedure was repeated six times per session, three sessions a week on alternate days (adapted from [24]).

The training program of VOT and AOT groups was based on the principle of overload with numbers of repetitions and rest similar to training in humans. The initial load was established as 75% of the body weight of each animal. Each week the animals were weighed (Oasus scales) for correcting the initial load in case there was any weight change. The adjustment of increment of the initial loads to promote training progression was done biweekly, always maintaining 75% of the weight updated, with 10% increases every two weeks for a progressive increase in the training load. Rats were trained three times per week for 12 weeks. Each animal had to climb the ladder to reach the top for six times on each training day, with approximately a minute interval between climbs. The resistance training was of moderate intensity (adapted from [24]).

Animals from the CVS, CAS, VOS, and AOS groups performed the exercises once a week without overload until they were submitted to euthanasia in order to trigger a stress similar to the VOT and AOT groups. After the intervention period, the animals were euthanized for the analyses of study parameters.

### 2.3. Diets Protocol

Since weaning until 14 months of age, the animals were fed with specific chow for laboratory mice (following the recommendations of the National Research Council and National Institute of Health, USA, NRC, 2011) of the Nuvital brand (NUVILAB CR1, produced by Nuvital Nutrients LTDA, Curitiba, PR), which is composed of 52% carbohydrates, 21% protein, and 4% lipids. The chow ingredients are of vegetable origin, namely, ground whole corn, soybean meal, wheat bran, calcium carbonate, dicalcium phosphate, sodium chloride, and vitamin mineral premix.

From the age of 14 months, the CVS, VOS, and VOT groups were fed with the same chow, and the CAS, AOS, and AOT groups were fed with animal protein-based chow specially formulated for this study by the Rhoster Laboratory (Rhosterlab, Rhoster Ltda). The chow, made with milk protein (casein) in the same proportion as the initial chow, was introduced into the routine of animals simultaneously with the beginning of resistance training. The ingredients of this chow are the following: corn starch, casein (85% of the total protein source), soybean protein, sucrose, soy oil, calcium carbonate, dicalcium phosphate, sodium chloride, and vitamin mineral premix. For technological reasons of production, to obtain an optimum pellet texture, it was not possible to provide 100% of the dietary protein from animal sources therefore, including about 15% of vegetable protein. According to the National Research Council and National Institute of Health (USA) (NRC, 2011) recommendations, the animals had free access to the chow in hanging feeders.  Table 1 shows chemical composition of the diet.

The food consumption of the studied animals was measured by the weight difference between the chow offered and the remaining rest collected in the cage after 24 hours, obtaining the amount in grams consumed daily by the animals during the 12 weeks of intervention protocol. The mean values of macronutrients (carbohydrates, proteins, and lipids) in grams per day were calculated from the chemical composition of each chow offered, calculated proportionally by the total amount of chow consumed per day per animal.

### 2.4. Euthanasia of Animals

The euthanasia of the animals was performed at age of 17 months, the end of the 12-week period of physical training and specific diet (CVS, VOS, VOT, CAS, OSA, and AOT). The animals were in fasting for eight hours prior to euthanasia for blood collection for biochemical analysis. The animals were weighed and subsequently decapitated. Blood was collected in test tubes without anticoagulant, the hindlimb was dissected, and the right medial gastrocnemius muscle of the leg was collected. Every muscle was cross-sectioned equitably in proximal and distal thirds. The middle part resulting of cuts was processed for histological and histochemical analyses.

#### 2.4.1. Histological Processing

The processing of the collected material to carry out the morphological and immunohistochemical analysis was through two different fixation techniques:

- The first one is fixation in 10% formalin, followed by histological processing using five nonserial sections per animal for Picrosirius staining for visualization of collagen fibers [25].
- The second one is cryofixation by immersion of the material in liquid nitrogen to evidence by NADH-tr the different types of muscle fibers and thus allow analyses of parameters: (a) the cross-sectional area (CSA) for each muscle fiber type (I, IIA, and IIB); (b) volume density of muscle fiber types, and (c) interstitial volume density. For the tissue cuts, frozen pieces were placed in a cryostat with chamber maintained at −25°C for 30 minutes for the adaptation of tissue. Then, they were subjected to ten-micrometre thick sections, which were placed on glass slides. We collected five semiserial cuts of each muscle fragment, and, for every six cuts obtained in processing, five were disregarded.

### 2.5. Quantitative Analysis of Tissues

#### 2.5.1. Morphometric Analysis of the Light Microscope

The morphometry was performed on scanned images, semiautomatically, with specific software for quantitative analysis [26]. All images were converted to JPEG files to later apply the stereology technique [27], using the Image J software (version 1.47, National Institutes of Health; Collins, 2007).

#### 2.5.2. Volume Density of Collagen Fibers (Vv[col])

The slides were stained by the Picrosirius method and examined under a light microscope coupled to Polaroid filters. The analysis was performed in four fields (upper right, upper left, lower right, and lower left), in each of the five sections of each slide per animal. The analysis of volume densities was performed by stereology, with a test system of 266 points, using the Image J software.

### 2.6. Histochemical Procedures 

#### 2.6.1. Classification of Muscle Fibers by NADH-tr (Tetrazolium Reductase)

The classification of muscle fibers was carried out following the criteria of the NADH reductase technique (NADH-tr) [28–30]. The type IIB fibers, glycolytic (white fibers, fast twitch) are stained weakly, while the type I oxidative (red fibers, slow twitch), which have high mitochondrial activity, are heavily stained. With intermediate coloration, the type IIA fibers, glycolytic-oxidative (intermediate fibers), are emphasized [31].

#### 2.6.2. Cross-Sectional Area of Myocytes (CSA)

In slides stained by NADH-tr, the cross-sectional area of type I, IIA, and IIB muscle fibers, present in 20 photographs distributed in four fields selected at random, was determined on the scanned images with ×100 increase. The analysis was done on five nonserial sections of each animal muscle, by using the Axio Vision software.

#### 2.6.3. Volume Density of Myocytes and Interstitial (Vv[myo] and Vv[int])

Five nonserial sections per animal were used, and four fields of each cut were analyzed by the immunohistochemistry technique NADH-tr with ×100 increase, a test system with 165 points and use of the Image J software.

#### 2.6.4. Biochemistry

The analyses of biochemical parameters were performed according to techniques traditionally used in clinical laboratories [32] and performed in duplicate, following the best practices in clinical analysis.

- Serum glucose: the serum obtained after centrifugation of blood was used for analysis of glucose by the glucose oxidase method.
- Albumin concentration: It is by the bromocresol green method.
- These two biochemical parameters were obtained in the semiautomatic analyzer BIO-2000 (BIOPLUS) with Doles commercial kits (Doles Reagents), following the manufacturer's recommendations. The measurements were performed in the clinical analyses laboratory of the NEF-3 (Center for Pharmaceutical Studies-3) of Universidade São Judas Tadeu (USJT).
- It is creatine phosphokinase dosage (CPK), following the manufacturer's recommendations and with use of the ELITech EL200 trade kits (ELITech Group Vital Scientific) and the automated analyzer FLEXOR-EL200 (ELITech Group Vital Scientific), in the Clinical Analysis Laboratory of Faculdade de Medicina do ABC (FMABC).
- It is dosage of homocysteine and ultrasensitive C-reactive protein (us-CRP), by the chemiluminescence method, using IMMULITE 1000 commercial kits (DPC) and following the manufacturer's recommendations on the autoanalyzer device IMMULITE-1000 (Siemens). The measurements were performed in the Clinical Analysis Laboratory of Faculdade de Medicina do ABC (FMABC).

### 2.7. Statistical Analysis

The data for each variable analyzed were tabulated and separated by group (VS, VOS, VOT, AS, AOS, and AOT). Then, the mean and median and standard deviation were calculated and subsequently compared statistically by the one-way ANOVA. Comparisons were made between the means of the studied animal groups in each variable analyzed. After that, the post hoc Bonferroni test was applied to locate the differences found by analysis of variance. The significance level adopted was *p* < 0.05.

## 3. Results

After the 12-week intervention protocol with diet and/or exercise, the analysis of tissue was carried out, and the data of five animals per study group were computed. The results obtained are described below.

Regarding the average weight of animals, the VOS group showed higher body mass values (*p* < 0.05) than the CVS and AOS groups only at age of 6 months. The statistical similarity indicates a homogeneous body mass among the different groups of animals, regardless of ovariectomy, diet, or training to which they were submitted (Table 2).

Table 3 presents data on the average weight of visceral adipose tissue of animals according to the groups. A greater amount of visceral fat (*p* < 0.05) is observed in ovariectomized groups fed with animal protein (AOS and AOT) when compared to controls (CAS and CVS), regardless of training.

**Figure 1:**
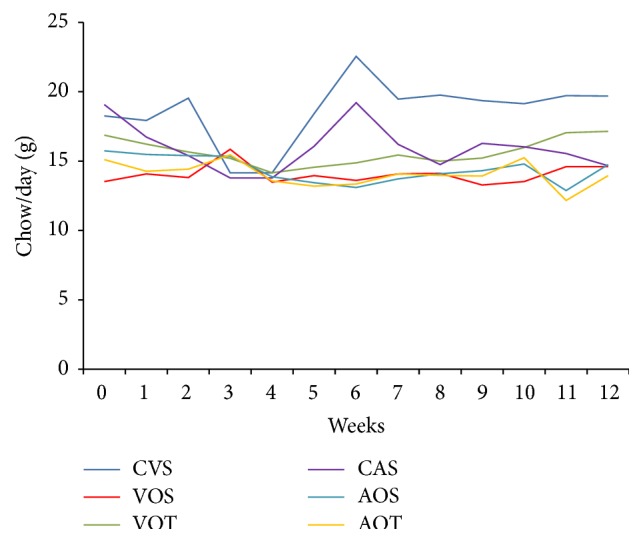
Average variation of food consumption (in grams of chow/animal/day), during the 12 weeks of intervention. Groups CVS, VOS, AOS, CAS, VOT, and AOT.

In relation to food intake, increased intake of chow and consequently of macronutrients and energy was observed, by the CVS group compared to the others (*p* < 0.05). The VOT and CAS groups also showed higher consumption compared to VOS, AOS, and AOT (*p* < 0.05) groups. Only the CVS group had higher mean than the other groups over time (*p* < 0.05), except in weeks 3 and 4 of the intervention protocol (Figure 1).

When the food intake was adjusted according to the animal body weight (Table 4), the CVS group had higher values (*p* < 0.05) of chow intake and consequently of macronutrients, compared to the mean of other groups.

### 3.1. Cross-Sectional Area (CSA) of Muscle Fibers

**Figure 2:**
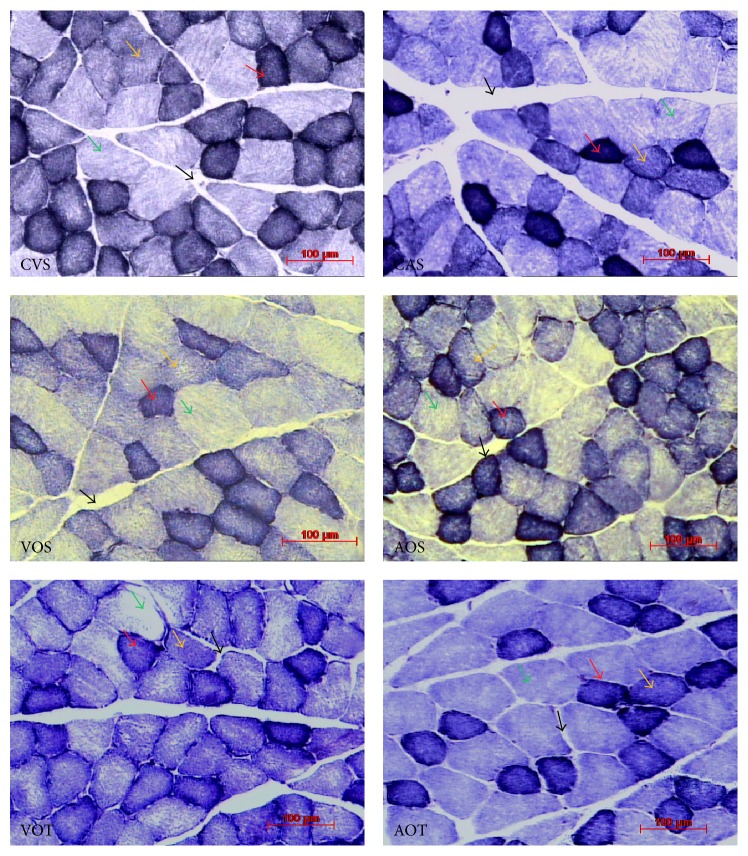
Cross-sectional images of the medial gastrocnemius muscle stained with the NADH-tr technique. CVS: sedentary, control, vegetable protein, VOS: sedentary, ovariectomized, vegetable protein, VOT: trained, ovariectomized, vegetable protein, CAS: sedentary, control, animal protein, AOS: sedentary, ovariectomized, animal protein, and AOT: trained, ovariectomized, animal protein. Arrows indicate the different types of fiber. Red: type I fibers, yellow: type IIA fibers, and green: type IIB fibers. Black arrows indicate interstitium.

The medial gastrocnemius muscle revealed fibers with polygonal contours and varying diameters, presenting mosaic distribution, and classified into three types; type I, type IIA, and type IIB. The results of the cross-sectional area of the medial gastrocnemius muscle myocytes are shown in Figure 2 and Table 5.

Analyzing the animals of the VOS group, smaller values (*p* < 0.05) of type I, IIA, and IIB fibers were observed when compared to control (CVS). When animals fed with animal proteins (AP) in the diet were analyzed, the data indicate significant effects (*p* < 0.05) of ovariectomy on the CSA of type I fibers in the AOS group (Table 5).

Only the type IIB fibers showed lower values (*p* < 0.05) compared to control (CAS), although the difference was of small magnitude. In the groups submitted to resistance training (AOT) there was a clear remodeling of the medial gastrocnemius muscle, since there was a significant reduction of the CSA of type I fibers (*p* < 0.05).

In these animals was also observed a reduction tendency (not significant) of the CSA of type IIA fibers and maintenance of values in type IIB fibers.

### 3.2. Volume Densities of Muscle Fibers (Vv[myo]) and Interstitium (Vv[int])

The results of the myocytes volume density (types I, IIA, and IIB) and the medial gastrocnemius muscle interstitium are shown in Table 6 and Figure 2.

Animals of the VOS group showed a decrease both in Vv[myo] of type I fibers and in Vv[int], when compared to the CVS group (*p* < 0.05). In the VOT group, there was no difference in Vv[myo] of the three fiber types, or in the interstitial space when compared to CVS group.

On the other hand, when the Vv[myo] and Vv[int] of animals in the AOS group were analyzed, there was an increase in Vv[myo] of type I fibers compared to the control group (AOS > CAS) (*p* < 0.05). In these same animals (AOS) was also observed a similar value for the type IIA and IIB fibers, compared to controls (AOS = CAS). Regarding the interstitium, data showed lower values in the AOS group compared to the CAS (*p* < 0.05).

Compared to the CAS group, the animals of the AOT group presented different values in Vv[myo] for the three fiber types (I, IIA, and IIB). The fibers of types I and IIA were smaller, and the type IIB fibers were larger than all other groups analyzed, including those who ingested vegetable proteins (*p* < 0.05).

In relation to Vv[int], the AOT group presented values similar to the control group (AOT = CAS) and larger values than the sedentary ovariectomized group (AOT > AOS; *p* < 0.05).

### 3.3. Volume Density of Collagen Fibers (Vv[col])

**Figure 3:**
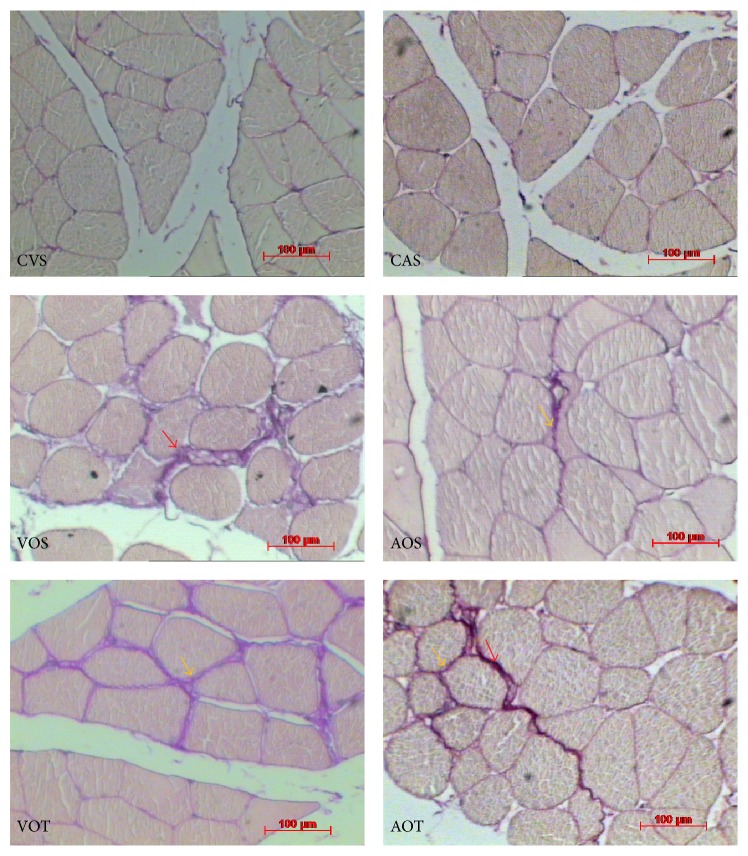
Cross-sectional images of the medial gastrocnemius muscle stained with the Picrosirius technique, showing the collagen fibers according to groups. CVS: sedentary, control, vegetable protein, VOS: sedentary, ovariectomized, vegetable protein, VOT: trained, ovariectomized, vegetable protein, CAS: sedentary, control, animal protein, AOS: sedentary, ovariectomized, animal protein, and AOT: trained, ovariectomized, animal protein. Arrows indicate collagen fibers stained with the Picrosirius method.

The analysis of the volume density of collagen fiber (Vv[col]) is presented in Table 7 and Figure 3.

The results showed similar Vv[col] values of the medial gastrocnemius muscle in animals of the CVS and CAS groups.

These same groups (CVS and CAS) had lower values than ovariectomized animals (CVS and CAS < VOS, VOT, AOS, and AOT; *p* < 0.05), regardless of training.

The animals submitted to resistance training showed higher values of Vv[col] than all other studied groups (*p* < 0.05).

### 3.4. Biochemical Parameters

There were no significant differences from a statistical point of view in blood glucose concentration in the animals analyzed, despite the trend towards higher blood glucose values in sedentary ovariectomized groups (+17.7% in the VOS and +17.5% in AOS) and lower values in trained ovariectomized groups (−27.3% in VOT and −28.7% in AOT), when compared to the respective controls. Regarding albumin concentrations the VOS group showed the lower values when compared with other groups (3.20 ± 0.60). Despite homocysteine concentrations, the CAS group showed the highest values (Table 8).

## 4. Discussion

In the present study, we observed a progressive and significant increase in body mass over the 17 months, regardless of ovariectomy, diet, and training to which the animals were subjected. Corroborating our results, Lushaj et al. [33] studied healthy Fisher rats and reported significant increase in body weight between five and 15 months. The interpretation of the study by Lushaj et al. [33] is that, in an animal model, both the body and muscle mass dramatically decrease only in very old animals.

There was a tendency to greater mass gain in animals subjected to ovariectomy, which is similar to the findings from several studies linking hormonal changes due to menopause with a mass gain especially of visceral adipose tissue [15, 34, 35].

Although there were no significant differences from a statistical point of view, the groups with animal protein diet showed a tendency to more mass gain and a greater final mass, when compared to the others. A possible explanation may be based on studies indicating that, compared to vegetable protein, the animal protein can promote greater muscle mass gain and hypertrophy in animals subjected to resistance training [36–40].

The chow offered to the studied animals had similar chemical composition in relation to macronutrients content. The only difference found was a higher fiber content of the chow with vegetable protein, given the nature of its composition ingredients (2.8% of fiber in the animal protein versus 8% in the vegetable protein). This higher fiber content may have been an important variable, interfering with the absorption of fat and other nutrients [41].

In this study, the distribution of collagen fibers and muscle fibers of type I (slow) and type II (IIA, intermediate, and IIB, fast) by volume density showed the distribution of collagen fibers with predominance of type IIB fibers, followed by type IIA and type I (type IIB: 50.87%; type IIA: 21.09%; and type I: 16.74%).

Unlike our findings, other authors have observed different distributions in the muscles of aged rats through immunohistochemical techniques. About 90–95% of the fibers of the vastus lateralis and the rectus femoris were of type II, while, in the medial vast, 80 to 85% were of type II [33]. In the present study, the muscles analyzed had approximately 70% of fibers histochemically classified as type II.

Our data are in agreement with studies by DeVries [42] and Borg and Caulfield [43], which used electron microscopy and showed differences between muscles of fast and slow contraction with respect to the intramuscular connective tissue arrangement of healthy muscles.

Because of this characteristic of rats, the protocol proposed in this study included the induction of sarcopenia through ovariectomy [44] in younger rats. Thus, it was possible to obtain significant degree of sarcopenia at an earlier age, when it is also more propitious to subject animals to progressive resistance training with heavy loads. This way, it was possible to analyze the effects of resistance training associated with diets with animal or vegetable protein in installed sarcopenia, even with a shorter period of experimentation (at 17 months of age).

Given the application of this protocol, one of the expected results was the occurrence of atrophy of the muscle fibers, characterized by decreasing CSA [33, 45]. Besides atrophy, another key aspect of sarcopenia expected as a result of ovariectomy is a decrease in the number of muscle fibers, as well as its replacement by connective tissue [46, 47]. In the present study, this characteristic is represented by the grouped analysis of volume density of myocytes, interstitium, and collagen fibers.

The association of the sarcopenia protocol and different diets showed that consumption of vegetable origin protein exclusively does not seem to be suitable for sedentary animals because, in this study, such consumption was not able to minimize the effects of sarcopenia in the morphological characteristics of muscles, especially in those of fast twitch, which tend to be most affected.

Resistance training associated with a vegetable protein diet, on the other hand, can be a helping factor (although with relative efficacy) for the inhibition or reversion of sarcopenia by promoting only a slight recovery of atrophy of fast glycolytic fibers, with concomitant increase of collagen support without, however, increasing interstitial density.

The medial gastrocnemius muscle of sedentary animals that consumed the animal protein diet had fewer effects of ovariectomy in the sarcopenia induction, compared to those who consumed the vegetable protein diet. The animals on animal protein diet showed hypertrophy of slow fibers, maintaining the intermediate fibers, and increased collagen with interstitial reduction. These changes characterize a more functional muscle [48], although still with atrophy (discrete) of fast fibers.

Moreover, when subjected to resistance training, the animals showed a significant muscle remodeling characterized by a reduction in volume density of slow and intermediate fibers and a significant increase in fast fibers [49]. This fact was accompanied by a significant increase in collagen density probably supporting collagen, since there was no increased interstitial density. In contrast, there was a tendency towards reducing density of interstitial volume, characteristic of a possible improved morphological profile, especially the gastrocnemius, which is essentially glycolytic muscle and important in activities that require fast twitch such as resistance training [50].

In terms of functional capacity, the effect of protein diet seems to have been more appropriate, even among sedentary animals, but this effect in functional capacity was more evident if associated with resistance training. This is justified due to the greater collagen density without interstitial increase, which is indicative of a greater support of IIB fibers, providing greater contractile strength and resistance to the fibers, which increased in volume density despite showing decreased CSA [51].

This greater volume density with smaller cross-sectional area is suggestive of a remodeling, with migration of fibers from the type IIA for the type IIB. This is justified by the reduced density of type IIA fibers, however without decreasing their area (i.e., without atrophy).

Finally, resistance training was unable to have a positive impact on reduction of adipose tissue or body weight of animals. Conversely, the body mass suffered substantial increase, influenced mainly by menopause and the animal protein diet. Factors such as menopause and exercise are better determinants of body composition than food consumption. The data suggest a trend towards better results in hypertrophy of the medial gastrocnemius muscle in the groups of animals who consumed the animal protein diet, even the sedentary, but more evident in trained animals, which is naturally responsive to the hypertrophy process when subjected to resistance training, since this is a predominantly glycolytic muscle.

## Figures and Tables

**Table 1:** Chemical composition of chow offered to the study animals per kg of product.

	Animal protein^*∗*^	Vegetal protein^*∗∗*^
	%	%
Moisture (max.)	7.60	12.50
Crude protein (min.)	22.5	22.0
Ether extract (max.)	4.80	4.00
Crude fiber (max.)	2.76	8.00
Mineral matter (max.)	7.32	9.00
Calcium (min.)	1.40	1.40
Phosphorus (min.)	0.80	0.80

Source: ^*∗*^Rhoster Ind e Com Ltda, Araçoiaba da Serra, SP. CNPJ 00 984 201/0001-04. ^*∗∗*^Nuvital. Colombo, PR. CNPJ 77 043 511/0001-15.

**Table 2:** Average body mass (g) and percentage variation of average body mass of the studied animals at age of 6, 14, and 17 months (mean ± standard deviation).

	Mass at 6 months (g)	Mass at 14 months (g)	Mass at 17 months (g)	Λ% from 6 to 14 months	Λ% from 14 to 17 months
CVS	217.00 ± 20.80	275.00 ± 17.68	300.00 ± 18.37	+26.73%	+9.09%
VOS	264.70 ± 13.62^*∗*^	248.13 ± 14.13	302.86 ± 48.72	−6.26%	+22.06%
VOT	252.50 ± 16.66	225.00 ± 16.69	348.57 ± 66.63	−10.89%	+54.92%
CAS	232.50 ± 6.45	279.00 ± 14.75	304.00 ± 16.73	+20.00%	+8.96%
AOS	226.40 ± 25.61	229.80 ± 17.20	353.67 ± 48.62	+1.50%	+53.90%^**∗∗**^
AOT	237.10 ± 18.22	227.50 ± 14.14	334.38 ± 46.40	−4.05%	+46.98%

^*∗*^VOS > VS and AOS (*p* < 0.05). ^*∗∗*^AOS > CAS (*p* < 0.05).

**Table 3:** Visceral adipose tissue mass (g), difference between the final body mass and the visceral adipose tissue mass (g) (FM-VAT), and percentage of adipose tissue in relation to body mass (%VAT) at the age of 17 months. Mean ± standard deviation.

Group	Mass VAT (g)	FM-VAT (g)	%VAT
CVS	11.12 ± 4.36^ab^	293.16 ± 16.39	3.56 ± 1.34^ab^
VOS	19.77 ± 14.43^a^	286.29 ± 38.09	5.03 ± 4.19^ab^
VOT	25.15 ± 15.55^a^	232.92 ± 51.25	6.60 ± 3.01^a^
CAS	11.56 ± 4.80^ac^	288.67 ± 18.40	3.78 ± 1.61^ab^
AOS	34.44 ± 12.52^ad^	319.52 ± 37.70	9.42 ± 2.29^ac^
AOT	34.65 ± 13.13^ad^	300.13 ± 34.17	9.94 ± 2.69^ac^

Equal letters represent equal averages; different letters represent the statistical differences (*p* < 0.05).

**Table 4:** Food consumption relative to body mass (in g of chow/g of animal body mass/day), carbohydrates, proteins, and lipids (in mg of nutrient/g of animal body mass/day) according to the group of study. Mean ± standard deviation.

	Chow (g/kg of body mass)	Carbohydrates (mg/g of body mass)	Proteins (mg/g of body mass)	Lipids (mg/g of body mass)
CVS	6.20 ± 0.45^**∗**^	0.0330 ± 0.0021^**∗**^	0.0140 ± 0.0009^**∗**^	0.0025 ± 0.0002^**∗**^
VOS	4.29 ± 0.95	0.0240 ± 0.0042	0.0102 ± 0.0018	0.0018 ± 0.0003
VOT	4.14 ± 1.07	0.0241 ± 0.0050	0.0102 ± 0.0021	0.0019 ± 0.0004
CAS	4.60 ± 0.55	0.0257 ± 0.0014	0.0109 ± 0.0006	0.0020 ± 0.0001
AOS	3.71 ± 0.76	0.0209 ± 0.0029	0.0089 ± 0.0012	0.0016 ± 0.0002
AOT	3.75 ± 0.71	0.0221 ± 0.0029	0.0093 ± 0.0012	0.0017 ± 0.0002

^**∗**^CVS ≠ from all the others (*p* < 0.05).

**Table 5:** Cross-sectional area of myocytes (*µ*m^2^) (CSA) of the medial gastrocnemius muscle according to fiber types. Mean ± standard deviation.

Group	Type I (*µ*m^2^)	Type IIA (*µ*m^2^)	Type IIB (*µ*m^2^)
CVS	2408.60 ± 631.89^a^	3260.64 ± 801.54^a^	4241.44 ± 1159.77^a^
VOS	2111.60 ± 642.29^b^	2744.56 ± 627.20^b^	4027.97 ± 1226.38^b^
VOT	1848.24 ± 872.50^c^	2652.21 ± 569.94^c^	4208.73 ± 1504.68^a^
CAS	2375.55 ± 535.07^a^	3074.60 ± 607.85^ab^	4151.06 ± 1129.33^c^
AOS	2660.12 ± 587.04^d^	3192.49 ± 787.13^b^	4079.76 ± 1268.78^a^
AOT	1741.67 ± 609.78^e^	2610.80 ± 859.44^ab^	4092.27 ± 1081.34^a^

Equal letters represent equal averages; different letters represent the statistical differences.

**Table 6:** Volume density (%) of type I, IIA, and IIB muscle fibers and interstitium of the medial gastrocnemius muscle. Mean ± standard deviation.

Group	Vv [type I fiber] %	Vv [type IIA fiber] %	Vv [type IIB fiber] %	Vv [interstitium] %
CVS	19.02 ± 7.78^ac^	23.38 ± 8.48^a^	48.25 ± 9.66^a^	9.09 ± 3.6^a^
VOS	13.54 ± 6.44^b^	27.00 ± 6.99^ac^	53.21 ± 8.53^ab^	5.57 ± 2.71^b^
VOT	18.37 ± 5.62^c^	26.73 ± 6.35^a^	45.74 ± 6.08^a^	8.90 ± 3.27^a^
CAS	15.38 ± 5.36^c^	21.62 ± 7.58^ad^	49.40 ± 9.62^a^	13.32 ± 3.62^c^
AOS	24.48 ± 7.06^d^	24.18 ± 6.53^a^	46.00 ± 7.02^ac^	4.65 ± 2.30^ab^
AOT	6.54 ± 3.76^e^	14.68 ± 4.76^b^	68.08 ± 10.99^d^	10.36 ± 8.18^ac^

Equal letters represent equal averages; different letters represent the statistical differences.

**Table 7:** Volume density of collagen fibers of the medial gastrocnemius muscle in the studied groups. Mean ± standard deviation.

Group	Vv [col] %
CVS	7.97 ± 4.73^a^
VOS	15.32 ± 5.58^b^
VOT	14.84 ± 3.53^b^
CAS	8.40 ± 4.20^a^
AOS	14.46 ± 8.28^b^
AOT	22.15 ± 7.33^c^
Total	14.53 ± 8.02^1^

Equal letters represent equal averages; different letters or numbers represent the statistical differences (*p* < 0.05).

**Table 8:** Serum concentrations of glucose, homocysteine, ultrasensitive C-reactive protein (CRP), and creatine phosphokinase (CPK) (mg/dL) and serum albumin (g/dL) according to animal groups. Mean ± standard deviation.

	Glucose (mg/dL)	Homocysteine (mg/dL)	us-CRP (mg/dL)	CPK (mg/dL)	Albumin (g/dL)
CVS	92.8 ± 15.16	11.16 ± 1.58^a^	0.54 ± 0.54	2710.00 ± 4048.98	4.41 ± 0.23
VOS	109.29 ± 33.05	8.04 ± 1.97^ab^	0.30 ± 0.00	3090.00 ± 2527.20	3.20 ± 0.60^*∗*^
VOT	92.7 ± 12.96	9.76 ± 3.29^a^	0.30 ± 0.00	2591.67 ± 2252.87	4.31 ± 0.34
CAS	125.40 ± 8.26	12.30 ± 1.03^ac^	0.55 ± 0.53	2700.00 ± 4006.15	4.79 ± 0.27
AOS	109.00 ± 39.74	8.99 ± 1.81^ab^	0.30 ± 0.00	1770.00 ± 1337.25	4.41 ± 0.72
AOT	66.75 ± 13.50	10.57 ± 1.48^a^	0.30 ± 0.00	683.33 ± 450.19	4.51 ± 0.32

^*∗*^VOS < than all the others. Equal letters represent equal averages; different letters represent the statistical differences (*p* < 0.05).
